# Fabrication and analysis of nanoemulsion-based edible films loaded with vitamin D3 and *Cordia myxa* mucilage

**DOI:** 10.1038/s41598-025-07214-z

**Published:** 2025-09-26

**Authors:** Parisa Rousta, Sedigheh Yazdanpanah, Mozhgan Shahamirian, Alireza Shirazinejad

**Affiliations:** 1 Department of Food Science and Technology, Sarv.C, Islamic Azad University, Sarvestan, Iran; 2 Department of Food Science and Technology, Kaz.C, Islamic Azad University, Kazerun, Iran; 3 Department of Chemistry, Faculty of Science, Sarv.C, Islamic Azad University, Sarvestan, 73451-173 Iran

**Keywords:** *Cordia myxa*, Vitamin D_3_, Nanoemulsion, Encapsulation, Film, Biophysics, Carbohydrates

## Abstract

This study developed a biodegradable nanoemulsion-based edible film containing encapsulated vitamin D3 and *Cordia myxa* (CM) mucilage to replace synthetic packaging. The mucilage showed strong antimicrobial activity against gram-positive bacteria, 67.4% antioxidant activity, and 218.2 µg/g flavonoid content. Nanoemulsions with various mucilage concentrations were characterized, and the best encapsulation efficiency (91.5%) was observed in the En1 sample. Films with encapsulated nanoemulsions demonstrated reduced water vapor permeability, lower solubility, improved thermal stability, and higher vitamin D3 retention over 14 days. FTIR analysis confirmed polysaccharides as the main component, supporting film biocompatibility. These findings suggest a promising, eco-friendly packaging solution using CM mucilage.

## Introduction

Recently, there has been growing interest in the development of active and biodegradable packaging materials as alternatives to conventional polymers, which are derived from non-renewable sources and pose environmental hazards^1^.

Active packaging technologies, which involve interactions between the packaging matrix, food components, and surrounding atmosphere, help to improve food safety, extend shelf life, and preserve the sensory and nutritional quality of food products^2,3^.

Among the various biopolymers used in edible films and coatings, polysaccharides are widely applied in the food industry due to their film-forming ability, biocompatibility, and structural versatility^4^. Carboxymethyl cellulose (CMC), for instance, is a well-established polysaccharide used in edible film production because of its transparency, flexibility, and mechanical strength. However, CMC films lack inherent antimicrobial and antioxidant activities^5^, prompting the incorporation of bioactive agents to enhance their functionality^6,7^.

One promising source of natural bioactive compounds is herbal mucilage, which exhibits strong antioxidant and antimicrobial properties attributed to its polyphenols, flavonoids, and phenolic acids^8,9^. In this regard, CM, a mucilaginous plant species native to tropical and subtropical regions, has attracted attention. The mucilage extracted from *Cordia myxa* fruit (CMF) peel is rich in polysaccharides such as arabinogalactans, uronic acids, and other hydrophilic compounds. Its structural chemistry—dominated by hydroxyl, carboxyl, and ester functional groups—confers unique characteristics including film-forming ability, emulsifying activity, viscosity enhancement, and moisture retention^10^. In traditional medicine, Cordia species have been used for their antibacterial, antiviral, and wound-healing effects due to their secondary metabolites including flavonoids (like rutin), terpenes, and alkaloids^11,12^. Despite these advantages, the potential of CM mucilage in active edible film production remains underexplored.

Another critical nutritional concern is vitamin D deficiency, which contributes to skeletal and nonskeletal disorders such as rickets, osteomalacia, cardiovascular disease, and autoimmune conditions^13^. Vitamin D3 (cholecalciferol), a fat-soluble vitamin, is poorly soluble in water, making its direct incorporation into hydrophilic film matrices challenging. However, nanoemulsions offer a viable delivery system, enabling better dispersion, protection against degradation (oxidative, thermal, photolytic), and controlled release of the vitamin^14–16^.

The novelty of this study lies in the development of a nanoemulsion-based edible film incorporating encapsulated vitamin D3 and CM mucilage, aiming to combine the bioactive properties of both components and improve the physicochemical and functional properties of the film. The objectives of this study were to: (i) evaluate the antimicrobial, antioxidant, and phytochemical properties of CMF peel mucilage; (ii) formulate and characterize nanoemulsions containing free and encapsulated vitamin D3; and (iii) develop and analyze CMC-based edible films enriched with these nanoemulsions in terms of structural, barrier, thermal, and release properties.

## Materials and methods

### Materials and reagent

Unsaturated sunflower oil and CMF were purchased from the local market (Firouzabad, Iran). Vitamin D_3_ was purchased from Sigma-Aldrich Co. Sodium chloride, calcium alginate and Tween 80, Mueller Hinton Broth and Agar were obtained from Merck, Germany. Microbial strains acquired from stock culture collection in the Shiraz medical science laboratory.

### Analysis of CMFP mucilage

#### Extraction

The peels of CMF were meticulously cleaned and peeled. Subsequently, the peels were dried at 55 °C for 5 h in a vacuum oven (BINDER, Germany). Once dried, the peels were ground using a mill and sieved through a 40-mesh sieve (IKA, Germany) to obtain a fine powder. To investigate the optimal solvent composition for extracting phytocompounds, various ethanol-to-water ratios of 30:70, 50:50, and 70:30 were employed. The extraction process was conducted using an ultrasonic homogenizer equipped with a probe (HD3200, Berlin, Germany), operating at a frequency of 24 kHz and a constant power output of 200 W for 50 min at a temperature of 25 °C. Following extraction, the liquid extract was separated from the solid residue by filtration through Whatman No. 1 filter paper. The solvent was subsequently removed from the extract using a rotary evaporator (VT-RT-EV-01, IRI). The resulting freeze-dried CMF powder was stored in a completely sealed plastic bag and maintained in a freezer at − 20 °C for further analysis^17^.

#### Antimicrobial activity

In order to activate the microbial strains Mueller Hinton Broth was used for 24 h at 37 °C. Then they were taken to Muller Hinton Agar medium and cultured linearly. Antimicrobial activity was tested against Gram-positive positive (*Bacillus cereus* (ATCC 11778) and *Staphylococcus aureus* (ATCC 6538) and Gram-negative (*Pseudomonas aeruginosa* (ATCC 10145), *Escherichia coli* (ATCC 35218), *Klebsiella pneumonia* (ATCC 13883), *Salmonella typhimurium*(ATCC 14028) bacteria. Subsequently, 5 mm diameter wells were established at the center of the Petri dishes, into which 20% of the samples were transferred. The Petri dishes were then incubated overnight at 37 °C in an incubator. The resulting diameter of the growth inhibition zone surrounding each well was measured and reported in millimeters^18^.

#### Antioxidant properties by DPPH

The DPPH assay was employed to evaluate the free radical inhibition effect (Pękal et al., 2015). In the presence of phenolic groups, DPPH readily undergoes radicalization. To conduct the assay, 0.1 mL of varying concentrations (5, 25, 65, 125, 250 µg/mL) of CMFP mucilage was combined with 0.9 mL of a 0.15 mM DPPH solution in methanol. The mixtures were kept in the dark at room temperature for 30 min. Absorbance measurements were then taken at 517 nm using a spectrophotometer (Schimadzu UV/Vis-240 IPC). The results were expressed as mg Trolox equivalents (TE)/100 g FW of the sample^19^.

$$\% {\text{scavenging}}\,{\text{activity}} = \left( {{\text{A}}0{-}{\text{A/A}}0} \right)$$

A0: control absorption, A: sample absorption.

#### Measurement of total flavonoid content

Flavonoid content was measured using the aluminum chloride colorimetric method (Babot et al., 2021). In this method, 0.5 mL of CMFP mucilage was mixed with 1.5 mL of 95% ethanol, 0.1 mL of 10% aluminum chloride, 0.1 mL of potassium acetate, and 2.8 mL of distilled water. The absorbance of the mixture was measured at a wavelength of 415 nm after 30 min at 25 °C. To construct a standard curve, quercetin was used in dilutions of 0, 20, 40, 60, 80, and 100 mg/L. The results were expressed as milligrams of quercetin equivalents per gram of mucilage (mg QE/g mucilage )^20^.

### Analysis of nanoemulsion

#### Production of free and encapsulated nanoemulsion

A 1% mucilage stock solution was prepared by dissolving the mucilage powder in double-distilled water (w/v). For nanoemulsion preparation, appropriate volumes of the mucilage stock were diluted to reach final concentrations of 0.1% and 0.2% (w/v) in the aqueous phase. The oil phase, containing sunflower oil enriched with vitamin D3 (at either 0.05% or 0.15%), was gradually added to the aqueous phase. Tween 80 (1.5%) was used as an emulsifier. The mixture was homogenized using an ultrasonicator equipped with a titanium probe (HD3200, Berlin, Germany) for 1 min at 10,000 rpm. For encapsulation, a 2% (w/v) hydrated calcium alginate solution was prepared and stirred for 30 min. Then, 0.5 g of the nanoemulsion containing mucilage was added to the alginate solution and pre-homogenized at 13,500 rpm for 1 min. Finally, the mixture was sonicated at 150 W and 20 kHz for 5 min at 25 °C using the same ultrasonic device. The temperature was maintained at 25 °C using an ice bath to preserve sensitive compounds such as vitamin D3^21,22^.

**Table 1:** Tested treatments of free and encapsulated nanoemulsion.

Sample^*^	Mucilage solution %	Double distilled water (%)	Sunflower oil (%)	Vitamin D_3_ (%)	Tween 80 (%)
C	–	75	23.5	–	1.5
N%0.1-%15D_3_	0.1	75	23.25	0.15	1.5
N%0.2-%5D_3_	0.2	75	23.25	0.05	1.5
En%0.1-%15D_3_	0.1	75	23.25	0.15	1.5
En%0.2-%5D_3_	0.2	75	23.25	0.05	1.5

^*^ C: control, N: Free Nanoemulsion, En: Encapsulated nanoemulsion.

### Characterization of nanoemulsions

The particle size distributions and ζ-potentials of both free and encapsulated nanoemulsions were determined using dynamic light scattering (DLS) measurements (Malvern Instruments Ltd., Malvern, UK). The DLS analyses were conducted at a wavelength of 633 nm, with scattering intensity recorded by a photodiode detector positioned at a 173° angle relative to backscattering with dilutions of (2, 6, 10%) to prevent multiple scattering effects^23^.

#### Measurement of pH and turbidity

To measure pH of for free and encapsulated nanoemulsion samples a digital pH meter (METROHEM company, Switzerland) was used, and the measurement was done at the temperature of 25 °C. Then, the turbidity of free and encapsulated nanoemulsion at a wavelength of 600 nm at 25 °C ( Schimadzu UV/Vis-240IPC) was measured by using a spectrophotometer^24^.

#### Encapsulation efficiency

1 ml of each encapsulated sample centrifuged for 30 min at 13,000 rpm (LABSCO, Germany) to separate the capsules. Then the samples were filtered by the use of 0.22 μm syringe head filter (Analisis Vínicos S.L, Tomelloso, Spain). Next, 2 ml methanol was used to dilute 40 µl of each isolated sample. Afterwards, the absorbance was read by a spectrophotometer (Schimadzu UV/Vis-240IPC) at the wavelength of 375 nm^25^. The calculation was done based on the following Eq. (1):

1 $${\text{EE}}\% = \left( {{\text{Total}}\,{\text{N}}{-}{\text{Free}}\,{\text{N}}/{\text{Total }}} \right)$$

Total N: total nanoemulsion used. Free N: free nanoemulsion. EE: microencapsulation efficiency.

### Analysis of edible film

#### Edible film production

A 1% w/v solution of carboxymethyl cellulose (CMC) was prepared. Glycerol was then added as a plasticizer at a concentration of 0.75 w/w. The mixture was stirred at 12,000 rpm for 10 min at 80 °C^26^. After cooling the solution to 37 °C, 20 mL of either free or encapsulated nanoemulsions were incorporated into the hydrated CMC solution. To homogenize the mixture, it was stirred at 500 rpm using a mixer for 30 min at 40 °C. A vacuum pump was used under ambient conditions for 5 min to remove air from the film-forming solution. Subsequently, 100 mL of each solution was poured onto smooth laboratory plates and allowed to dry at 25 °C^6^. A control sample without nanoemulsion was also prepared.

#### Apparent viscosity

Two hundred milliliters of each film-forming solution were measured for apparent viscosity using a rotary viscometer (BROOKFIELD, USA). The measurements were taken by varying the rotational speed to 100, 150, and 200 rpm at 25 °C with spindle number 1^27^.

#### Microstructure

The scanning electron microscope (TESCAN vega3, Czech Republic) was used to study the surface of the film samples at a power of 5 kW. Small pieces of the film samples were attached to the aluminum support base using silver glue. The samples were first coated with gold for 5 min in a coating/spraying device (DSR1, Nanostructured Coating Company, Iran) and then photographed^26^.

#### Surface color of films

A digital camera (Canon A540, 6 megapixels) was used in a wooden box with dimensions of 0.5 × 0.5 ×  0.6 m^2^ to measure the color of the produced films^28^. The collected data were analyzed using Adobe Photoshop^®^ CS5. Parameters such as L*, a*, and b*, which indicate brightness, red-green, and yellow-blue respectively, were also calculated^5^

#### Permeability to water vapor

To prepare 0% RH, aluminum cups were filled with 50 g of CaCl_2_, and the cups were covered with a sample of film containing free and encapsulated nanoemulsion and kept in a desiccator with saturated sodium chloride (NaCl) solution at 20 °C (RH75%). Then, each of the cups was weighed with an interval of 1–24 h to determine the permeability to water vapor, Eq. (4) was calculated (g/h mm kpa)^29^.

$$\:\text{W}\text{V}\text{P}=\frac{WVTR\:\times\:T}{P0\:\times\:S\:\times\:(\text{R}\text{H}\text{o}\:-RHi)}$$

T: the average thickness of film samples (mm). P0: saturated water vapor pressure at 20 °C (Pa). S: areal surface (cup) of the film (mm^2^). RHo -RHi: the difference in anhydrous calcium chloride and saturated sodium chloride solution at 20 °C.

#### Solubility in water

The pieces of film (1 × 4 cm²) were placed in an oven (IKA Oven 125 basic dry, Germany) at 105 °C for 24 h to reach a constant weight. After that, the dried films were immersed in 50 mL of double-distilled water (DDW) at a temperature of 25 °C for 6 h and stirred at a speed of 100 rpm. Then, the pieces of film were separated from the water. After drying at 50 °C for 24 h, they were weighed. The solubility percentage of the films was calculated using Eq. (2)^30^.

2 $$\% {\text{ Solubility}} = {\text{w1}} - {\text{w2}}/{\text{w1}} \times 100$$

w1 = initial dry weight. w2 = final dry weight.

#### Thickness

The thickness of the produced films was measured with a digital micrometer (Mitutoyo, Tokyo, Japan) to an accuracy of 1 μm. Measurements were taken from at least ten random locations on each sample^31^.

#### Density

The density of the film (g/mm³) was calculated by dividing the film’s weight (g) by its volume (mm³). The volume was determined by multiplying the surface area of the film (mm²) by its thickness (mm)^31^.

$${\text{Density}}\left( {{\text{g}}/{\text{cm}}^{{\text{3}}} } \right) = {\text{weight}}\,{\text{of}}\,{\text{sample}}\left( {\text{g}} \right)/{\text{volume}}\,{\text{of}}\,{\text{sample}}\left( {{\text{cm}}^{{\text{3}}} } \right)$$

#### Vitamin D_3_ stability

To determine the vitamin content, measurements were taken on days 0, 7, and 14. One gram of each film, containing either free or encapsulated nanoemulsion, was added to 9 g of ethanol and stirred until the vitamin D3 was fully extracted. The mixture was then centrifuged at 1750 rpm for 15 min at 4 °C (LABSCO, Germany). The supernatant was analyzed at 265 nm using a spectrophotometer (Schimadzu UV/Vis-240 IPC)^32^.

#### FTIR spectroscopy

To perform the FTIR test, a Spectrum Two device (FTIR Perkin Elmer, USA) was used. Thin tablets of film samples, containing both free and encapsulated nanoemulsion, were prepared with a thickness of less than 1 mm by mixing the samples with water and coating them with KBr, then freeze-drying at − 70 °C (Operon, Korea). The tablets were formed at a ratio of 1:20 by applying a pressure of approximately 60 kPa for 10 min in a tablet press machine. The transmission spectrum of the samples was analyzed in the range of 400–4000 cm⁻¹ with a resolution of 5 cm⁻¹^33^.

#### Thermal gravimetric analysis (TGA)

TGA analysis of the film samples was performed under a nitrogen atmosphere using a Thermogravimetric Analyzer (NETZSCH, 2000 F3 Maia, Germany) and also confirmed with a Perkin Elmer TGA 4000 (Germany). The device was calibrated with indium^34^. Samples of free and encapsulated nanoemulsion films, weighing approximately 4 mg each, were placed in an aluminum pan and heated at a rate of 10 °C/min from 0 to 500 °C, over a period of 45 min^35^.

### Statistical analysis

The measurements were conducted and repeated three times. The results were reported as mean ± standard deviation (SD). Analysis of variance (ANOVA) was performed, and significant differences between mean values were determined using Duncan’s multiple range test with a significance level of *P* < 0.05. Statistical analysis was carried out using SAS software (version 9.1, SAS Institute Inc., Cary, NC).

## Result and discussion

### Antimicrobial effect of CMF Peel mucilage

The antimicrobial potential of CMF peel mucilage was evaluated against six foodborne pathogenic bacteria using the agar well diffusion method. The results, expressed as the diameter of inhibition zones (mm ± SD), are presented in Table 2. CMF peel mucilage exhibited varying degrees of antimicrobial activity depending on the bacterial strain. The most significant inhibition was observed against gram-positive bacteria, particularly *Bacillus cereus* (29.00 ± 0.70 mm) and *Staphylococcus aureus* (26.00 ± 0.60 mm). These strong effects may be attributed to the more permeable peptidoglycan-rich cell walls of gram-positive bacteria, which are more susceptible to the bioactive compounds in plant mucilage. In contrast, gram-negative bacteria such as *Salmonella typhi* (7.00 ± 0.35 mm) and *Pseudomonas aeruginosa* (9.00 ± 0.40 mm) showed the least sensitivity, likely due to their outer membrane barriers and efflux mechanisms. Moderate activity was seen against *E. coli* (18.00 ± 0.45 mm) and *Klebsiella pneumoniae* (16.00 ± 0.58 mm). These results align with previous findings. Al-Musawi et al. (2022) reported inhibition zones ranging from 13.3 to 17.5 mm for various pathogens treated with CM ethanolic extract, confirming the broad-spectrum antimicrobial nature of this plant. Similarly, other studies have demonstrated that plant mucilage can inhibit bacterial growth through the presence of phenolic compounds, flavonoids, and alcohol derivatives^36,37^. Key compounds such as mono (2-ethylhexyl) phthalate, alpha-tocopherol, phytol, stigmasterol, and neophytadiene have been identified as contributors to this bioactivity^38^.

The observed differences in inhibition zones may reflect variations in the concentration and nature of these phytochemicals in the CMF peel mucilage. A positive correlation has been suggested between phenolic content and antimicrobial strength^39^, supporting the potential use of CMF peel mucilage as a natural antimicrobial agent in edible film formulations.

**Table 2:** Antimicrobial effect of CMF Peel mucilage against pathogenic bacteria.

Sample	Bacillus cereus	Staphylococcus aureus	Pseudomonas aeruginosa	E. coli	Klebsiella pneumoniae	Salmonella typhi
	Zone of inhibition (mm ± SD)					
CMF Peel Mucilage	29 ± 0.7	26 ± 0.6	9 ± 0.4	18 ± 0.45	16 ± 0.58	7 ± 0.35

### Antioxidant activity of mucilage

The antioxidant activity of CMF peel mucilage was determined based on DPPH radical scavenging capacity, which was found to be 67.42%, alongside a total flavonoid content of 218.16 µg/g. These results highlight the promising potential of CMF peel mucilage as a natural antioxidant agent in functional food and packaging applications. The antioxidant effect is primarily attributed to the presence of phenolic and flavonoid compounds within the mucilage. These compounds can act as hydrogen or electron donors, effectively neutralizing free radicals such as DPPH. The hydroxyl (-OH) groups in phenolics are known to contribute to this radical scavenging mechanism, enhancing the bioactivity of the mucilage matrix.

In this study, hydrocolloids were extracted using a 70% ethanol–water solution combined with ultrasound-assisted extraction, which significantly enhanced the yield of antioxidant compounds. Ethanol’s moderate polarity reduces the dielectric constant of the solvent mixture, facilitating the dissolution and diffusion of phenolic components^40^. Furthermore, the ultrasonic treatment likely promoted cavitation, disrupting cell walls and improving mass transfer, thereby increasing the extraction efficiency of flavonoids^41,42^. Flavonoid contents of 2–8 mg QE/g in lemon extracts have been reported using similar ethanol–water extraction methods, supporting the validity of this approach. The relatively high flavonoid concentration in the current study is consistent with the observed antioxidant activity, reinforcing the positive correlation between total flavonoids and DPPH scavenging activity^39,43^. These findings suggest that CMF peel mucilage possesses significant antioxidant capacity and could serve as an effective natural additive in the development of active edible films.

### Analysis of free and encapsulated nanoemulsion

#### Particle size and zeta potential

**Fig. 1:**
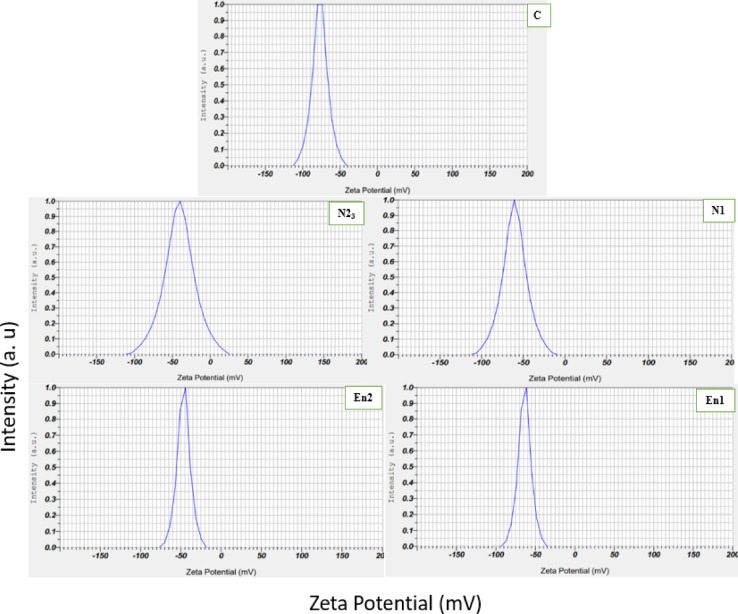
Particle size distribution curve of free and encapsulated nanoemulsion.

Particle size and zeta potential are critical parameters influencing the stability, dispersion behavior, and bioavailability of nanoemulsions. As shown in Table 3, significant differences (*p* < 0.05) were observed among all samples in terms of both particle size and zeta potential. The largest droplet size was found in the free nanoemulsion sample N2 (3231.7 ± 0.10 nm), while the smallest size was observed in the encapsulated sample EN1 (1273.5 ± 0.67 nm). In general, encapsulated nanoemulsions had smaller particle sizes compared to their free counterparts. This reduction can be attributed to the stabilizing effect of encapsulation, which restricts droplet coalescence. However, with increasing CMFP mucilage concentration, particle size tended to increase—likely due to higher viscosity of the continuous phase, which can impede efficient droplet breakup during emulsification^44^. The zeta potential (ζ) values also differed significantly among treatments (*p* < 0.05). The highest negative zeta potential was recorded in sample N1 (− 75.50 ± 0.10 mV), while the least negative value was observed in EN2 (− 0.40 ± 0.10 mV). A high absolute zeta potential value is typically associated with better colloidal stability due to enhanced electrostatic repulsion^45^. The observed differences between free and encapsulated nanoemulsions suggest potential interactions between the mucilage matrix and the nanoemulsion droplets, affecting their surface charge. Interestingly, increasing vitamin D3 concentration led to a more negative zeta potential, indicating that molecular interactions involving vitamin D3 could influence the surface charge and potentially enhance stability. These findings are supported by Fig. 1, which displays single-peaked size distribution curves, confirming the monodispersity of all formulations. Moreover, higher vitamin D3 concentrations yielded narrower and more elongated distributions, particularly in the encapsulated EN1 sample, reflecting better control over particle size uniformity. These results are in line with previous studies. Citrus oil nanoemulsions with droplet sizes of 34.23 nm and zeta potentials of 25.87 mV have been reported^46^, while dihydromyricetin-loaded emulsions showed droplet sizes of 56.54 ± 0.36 nm and zeta potentials of − 5.21 ± 0.54 mV^47^. Lipid composition has also been shown to significantly affect droplet size in vitamin C-loaded nanoliposomes^48^.

**Table 3:** Particle size and zeta potential of free and encapsulated nanoemulsion.

Name	Sample	Zeta(mV)	Size (nm)
C	(250ppmD_3_)	0.10^b^ ± -50.60	0.10^d^ ± 1631.1
N1	N100ppm-150ppmD_3_	0.10^e^ ± -75.50	0.10^b^ ± 2290.7
N2	N200ppm-50ppmD_3_	0.10^c^ ± -59.10	0.10^a^ ± 3231.7
En1	En100ppm-150ppmD_3_	0.10^d^ ± 60.50-	0.67^e^± 1273.5
En2	En200ppm50ppmD_3_	0.10^a^ ± -10.40	0.10^c^ ± 1895.2

Different letters in the same column indicate a significant difference among the samples, as revealed by Duncan’s test, *p* < 0.05.

#### pH, turbidity and efficiency of encapsulation

One of the conditions that stabilizes the emulsion system and affects the zeta potential is the optimal pH condition^49^. The findings presented in Table 4 indicated that the N1 and N2 samples exhibited the lowest pH level and highest turbidity level, with no statistically significant difference (*p* > 0.05). Conversely, the control, En1 and En2 samples, displayed the highest pH level, without a statistically significant difference (*p* > 0.05). The lowest turbidity related to control sample with 250 ppm of vitamin D_3_ that shows the positive effect of vitamin D_3_ in the reduction of the turbidity^47^.

pH and turbidity are key parameters influencing the physicochemical stability of nanoemulsions. As shown in Table 4, samples N1 and N2 (free nanoemulsions) exhibited the lowest pH values (7.11 ± 0.01 and 7.13 ± 0.01, respectively) and highest turbidity values (1.965 ± 0.01 and 2.058 ± 0.01), while control (C), EN1, and EN2 demonstrated significantly higher pH and lower turbidity (*p* < 0.05). This pattern suggests that encapsulation contributes to stabilizing the emulsion system, possibly by limiting particle aggregation and reducing light scattering. The control sample (250 ppm vitamin D3) exhibited the lowest turbidity (0.401 ± 0.15), indicating the role of vitamin D3 in enhancing system clarity^47^. Optimal pH conditions improve emulsion stability by affecting zeta potential and particle dispersion^49^. The increase in turbidity in N1 and N2 can be attributed to a decrease in pH, which facilitates complex formation among the emulsion components. This results in larger particle aggregates, contributing to higher turbidity levels. Conversely, increasing vitamin D3 concentrations enhances electrostatic and steric repulsion, limiting particle interaction and reducing turbidity^50^. Regarding encapsulation efficiency, EN1 and EN2 samples achieved 91.5% and 91%, respectively. These high values reflect the effective encapsulation capacity of CMF peel mucilage, which provides an optimal matrix for trapping vitamin D3. Factors influencing encapsulation efficiency include the method of preparation, surface characteristics of the carriers, and the volume-to-surface ratio of the nanoemulsion droplets^51^. Nano-phytosomes with an encapsulation rate of 98%, attributed to adequate lecithin coverage, have also been reported in previous studies^52^. In the present study, the results confirm the suitability of CMF peel mucilage as a natural encapsulating agent with high efficiency and stability.

**Table 4:** pH, turbidity and encapsulation efficiency.

Name		Sample		Efficiency %		Turbidity		pH	
C		C(250ppmD_3_)		^−^		0.015^d^ ±0.401		0.15^a^ ± 7.77	
N1		N100ppm-150ppmD_3_		^−^		0.01^a^ ± 1.965		0.01^b^ ± 7.11	
N2		N200ppm-50ppmD_3_		^−^		± 0.01^a^ 2.058		± 0.01^b^ 7.13	
En1		En100ppm-150ppmD_3_		91.5^a^		0.01^c^ ± 0.954		0.10^a^ ± 7.61	
En2		En200ppm50ppmD_3_		91^b^		1.646 ± 0.01^b^		7.76 ± 0.11^a^	

Different letters in the same column indicate a significant difference among the samples, as revealed by Duncan’s test, *p* < 0.05.

### Edible film tests

#### Apparent viscosity of the Film-Forming solution

The rheological behavior of the film-forming solutions (FFS) plays a key role in determining the final quality of edible films. As shown in Table 5, the highest viscosity was recorded in the control sample (C: 2494.2 mPa·s), while the lowest viscosity was observed in N2 (2456.1 ± 0.02 mPa·s), which showed no significant difference from EN1 (2457.2 ± 0.01 mPa·s) (*p* > 0.05). The viscosity values across samples were significantly affected by the type and concentration of mucilage and the encapsulation state (*p* < 0.05). In non-encapsulated samples (N1, N2), increasing the mucilage concentration decreased viscosity, likely due to disruption of network structure at higher concentrations. Conversely, in encapsulated samples (EN1, EN2), higher mucilage concentration led to increased viscosity, possibly due to enhanced droplet interaction and the presence of encapsulation matrix components that contribute to internal friction. From a formulation perspective, the viscosity of FFS must be optimized to ensure uniform casting without sagging or bubble formation. Highly viscous solutions can hinder proper spreading, while low-viscosity solutions may not provide sufficient film thickness or cohesion. Rheological properties depend on molecular structure, interactions, and dispersion behavior of biopolymers, especially in non-Newtonian systems, where viscosity is shear-rate dependent^53^. At lower shear rates, higher viscosity values were observed, which decreased with increasing shear rate due to shear-thinning behavior. This is associated with the breakdown of entangled mucilage chains, deformation of droplets, and weakening of hydrogen bonds^54,55^. Increased kinetic energy at higher shear rates also leads to reduced polymer chain interactions, contributing to lower resistance to flow^56^. Furthermore, the structure and thickness of the film layer, particularly in encapsulated systems, may influence viscosity due to phase entrapment and interactions between dispersed droplets and the continuous matrix^57^. These findings underline the importance of controlling rheological properties for achieving optimal film-forming characteristics.

**Table 5:** The rheological properties of film forming solution.

Name		Sample		Torque mN·m		Viscosity mPa·s			Shear stress Pa		Shear rate 1/s
C		C(250ppmD_3_**)**		0.01^e^ ± 0.9611		1.01^a^ ± 2494.2			0.1^a^ ± 74.51		0.6^c^ ± 50.50
N1		N100ppm-150ppmD_3_		0.01^a^ ± 1.96		1.03^b^ ± 2489.2			0.1^b^ ± 73.15		0.6^b^ ± 51.01
N2		N200ppm-50ppmD_3_		0.07^c^ ± 0.9670		1.02^d^ ± 2456.1			0.1^c^ ± 72.15		0.6^c^ ± 50.60
En1		En100ppm-150ppmD_3_		0^d^ ± 0.9634		1.01^d^ ± 2457.2			0.7^c^ ± 72.15		2.2^d^ ± 51.05
En2		En200ppm50ppmD_3_		0^b^ ± 0.9736		1.01^c^ ± 2468.1			0.1^b^ ± 73.15		0.1^a^ ± 52.05

Different letters in the same column indicate a significant difference among the samples, as revealed by Duncan’s test, *p* < 0.05.

#### Microstructure of film

**Fig. 2:**
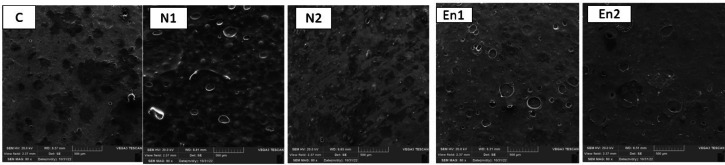
Morphological images of free and encapsulated nanoemulsion film.

The surface morphology of the edible films was examined using scanning electron microscopy (SEM), and the images are presented in Fig. 2. The results revealed that all films exhibited relatively uniform microstructures with good miscibility between film components. The matrix appeared continuous with well-distributed nanoemulsion particles, indicating efficient integration within the polymeric network. In the N1 treatment, the SEM images showed more spherical and interconnected pores, suggesting better internal structural uniformity. Compared to other samples, the porous structure was more developed, and the network of interconnected voids was clearly visible, reflecting effective film formation. Both free and encapsulated nanoemulsion-based films exhibited high internal density and low porosity, particularly when compared to the control (C) film. This reduction in voids implies stronger molecular packing and higher compatibility between the mucilage and the carboxymethyl cellulose (CMC) matrix. Increased vitamin D3 concentration resulted in greater surface roughness, which may be attributed to higher film thickness and nanoemulsion aggregation, as observed in previous studies^58^. The uniform dispersion of these nanoemulsions within the matrix also confirms the stabilizing role of mucilage, which enhances molecular interactions and contributes to structural integrity. Microcracks observed in some samples—particularly in EN1 and EN2—could be attributed to electrostatic damage during high-resolution imaging, as well as drying-induced stresses that lead to surface wrinkling^59^. Despite these imperfections, the films maintained coherent structures with minimal defects, and the surface porosity and roughness remained within acceptable limits for food-grade applications. Overall, SEM analysis confirmed the efficient incorporation of both free and encapsulated nanoemulsions within the CMC-based matrix and highlighted the favorable microstructural properties of the developed edible films.

#### Surface color

Surface color is an essential visual attribute for edible films, especially in applications where transparency and appearance are critical for consumer acceptance. As shown in Table 6, the color parameters L*, a*, b*, and ΔE were significantly affected by the type and concentration of mucilage and the presence of nanoemulsion (*p* < 0.05). The L* value, which reflects brightness (with 0 indicating black and 100 indicating white or transparency), was highest in the control sample (82.47 ± 0.06) and encapsulated treatments, indicating higher lightness and transparency. The lowest L* value was recorded in N2 (77.51 ± 0.17), suggesting that free nanoemulsion and higher mucilage concentrations slightly reduced transparency. The a* parameter, where positive values indicate redness and negative values indicate greenness, shifted toward lower (more negative) values with increasing mucilage concentration, reflecting a more greenish tone. However, increasing vitamin D3 content led to higher a* values, shifting the tone slightly toward red. These interactions between formulation components influenced the final hue of the films. Similarly, b* values, indicating yellow (+ b) or blue (-b), increased with higher mucilage content. The maximum b* value (14.34 ± 0.01) was seen in N2, indicating a more yellowish hue, which may be attributed to the presence of phenolic compounds naturally present in CM mucilage^60^. The total color difference (ΔE) was significantly higher in EN2 (31.99 ± 5.29), reflecting the combined effect of mucilage and vitamin D3 concentration on visual perception. Despite this, the incorporation of both free and encapsulated nanoemulsions did not cause undesirable visual changes to the films. All samples retained acceptable appearance characteristics suitable for food-grade packaging, consistent with findings from previous studies^28,61^.

**Table 6:** Surface color of free and encapsulated film with Hunterlab.

Sample	Name	L^*^	a^*^	b^*^	$\:\varDelta\:\varvec{E}$
(250ppmD_3_) C	C	0.06^a^ ± 82.47	0.01^a^± -3.16	0.07^e^± 10.28	0.06^b^ ± 29.65
N100ppm-150ppmD_3_	N1	0.10^d^ ± 78.70	0.01^b^ ± -6.15	0.03^c^ ± 13.24	0.10^c^± 28.59
N200ppm-50ppmD_3_	N2	0.17^e^ ± 77.51	0.01^c^ ± -7.12	0.01^a^± 14.34	0.10^d^ ± 28.24
En100ppm-150ppmD_3_	En1	0.00^c^ ± 79.39	0.01^b^ ± -6.19	0.18^d^± 12.45	1.65^e^± 25.77
En200ppm50ppmD_**3**_	En2	0.16^b^ ± 80.53	0.01^c^ ± -7.5	0.17^b^± 13.52	0.529^a^ ± 31.99

Different letters in the same column indicate a significant difference among the samples, as revealed by Duncan’s test, *p* < 0.05.

#### Antimicrobial activity

The antimicrobial properties of the developed edible films were evaluated against gram-positive and gram-negative bacteria, and the results are presented in Table 7. All formulations, including both free and encapsulated nanoemulsions, exhibited inhibitory effects on bacterial growth to varying degrees. Among the tested formulations, films containing free nanoemulsion (N1 and N2) demonstrated the strongest antimicrobial activity, especially against *Bacillus cereus* (5.50 ± 0.04 mm) and *Staphylococcus aureus* (5.50 ± 0.04 mm in N2). This enhanced activity may be attributed to the direct interaction of active compounds with microbial cells, as the absence of encapsulation allows for faster diffusion and greater bioavailability of the bioactive agents. Phytochemical compounds such as tannins, flavonoids, alkaloids, and other plant-derived secondary metabolites have also been reported to exert antimicrobial defense mechanisms against a wide range of microorganisms^62^. The presence of such compounds in the CMFP mucilage likely contributes to the observed antimicrobial effects. In contrast, encapsulated nanoemulsion films (EN1 and EN2) showed weaker inhibition zones, suggesting that the encapsulation matrix may delay or reduce the release of antimicrobial compounds. However, even encapsulated films demonstrated effective inhibition, particularly against *Staphylococcus aureusand* and *Bacillus cereus*, indicating that the release was sufficient to exert bacteriostatic effects. The control film (C) containing only vitamin D3 also exhibited moderate antimicrobial activity, likely due to the intrinsic antibacterial properties of vitamin D3 itself, as well as potential synergistic effects with the film matrix. In general, gram-positive bacteria, particularly *Staphylococcus aureus*, showed greater sensitivity compared to gram-negative strains like *Klebsiella pneumoniae* and *Salmonella typhi*. This is consistent with previous studies, which report that the thicker peptidoglycan layer of gram-positive bacteria allows for easier penetration of antimicrobial agents^58^. The findings are in agreement with previous reports demonstrating effective release of antimicrobial compounds from biopolymer films into the surrounding environment^63^. Similarly, it has been reported that while rosemary essential oil exhibited antimicrobial activity in its free form, its efficacy was further enhanced upon encapsulation in a starch–CMC double-layer film, especially against *Staphylococcus aureus*^64^.

**Table 7:** Antimicrobial effect of free and encapsulated nanoemulsion films.

Sample Zone of inhibition (mm ± SD)	Bacillus cereus	Staphylococcus aureus	Pseudomonas aeruginosa	*E. coli*	Klebsiella pneumoniae	Salmonella typhi
(250ppmD_3_) C	0.05^c^ ± 3.50	0.05^e^ ± 3.50	0.05^c^ ± 3.00	0.01^c^ ± 2.50	0.01^a^ ± 4.5	0.03^c^ ± 3.00
N100ppm-150ppmD_3_	0.05^a^ ± 4.50	0. 05^c^ ± 4.50	0.04^a^ ± 4.00	0.04^c^ ± 2.50	0.01^b^ ± 3.5	0.03^b^ ± 3.50
N200ppm-50ppmD_3_	0.03^a^ ± 4.50	0.04^a^ ± 5.50	0.05^a^ ± 4.00	0.05^a^ ± 4.00	0.05^a^ ± 4.5	0.008^a^ ± 4.50
En100ppm-150ppmD_3_	0. 06^b^ ± 4.00	0.07^b^ ± 5.17	0.03^b^ ± 3.50	0.05^b^ ± 3.00	0.02^d^ ± 2.00	0.01^d^ ± 2.50
En200ppm50ppmD_**3**_	0. 04^b^ ± 4.00	0.03^d^ ± 4.00	0.03^c^ ± 3.00	0.06^b^ ± 3.00	0.05^c^ ± 3.00	0.05^e^ ± 1.50

Different letters in the same column indicate a significant difference among the samples, as revealed by Duncan’s test, *p* < 0.05.

#### DPPH assay

**Fig. 3:**
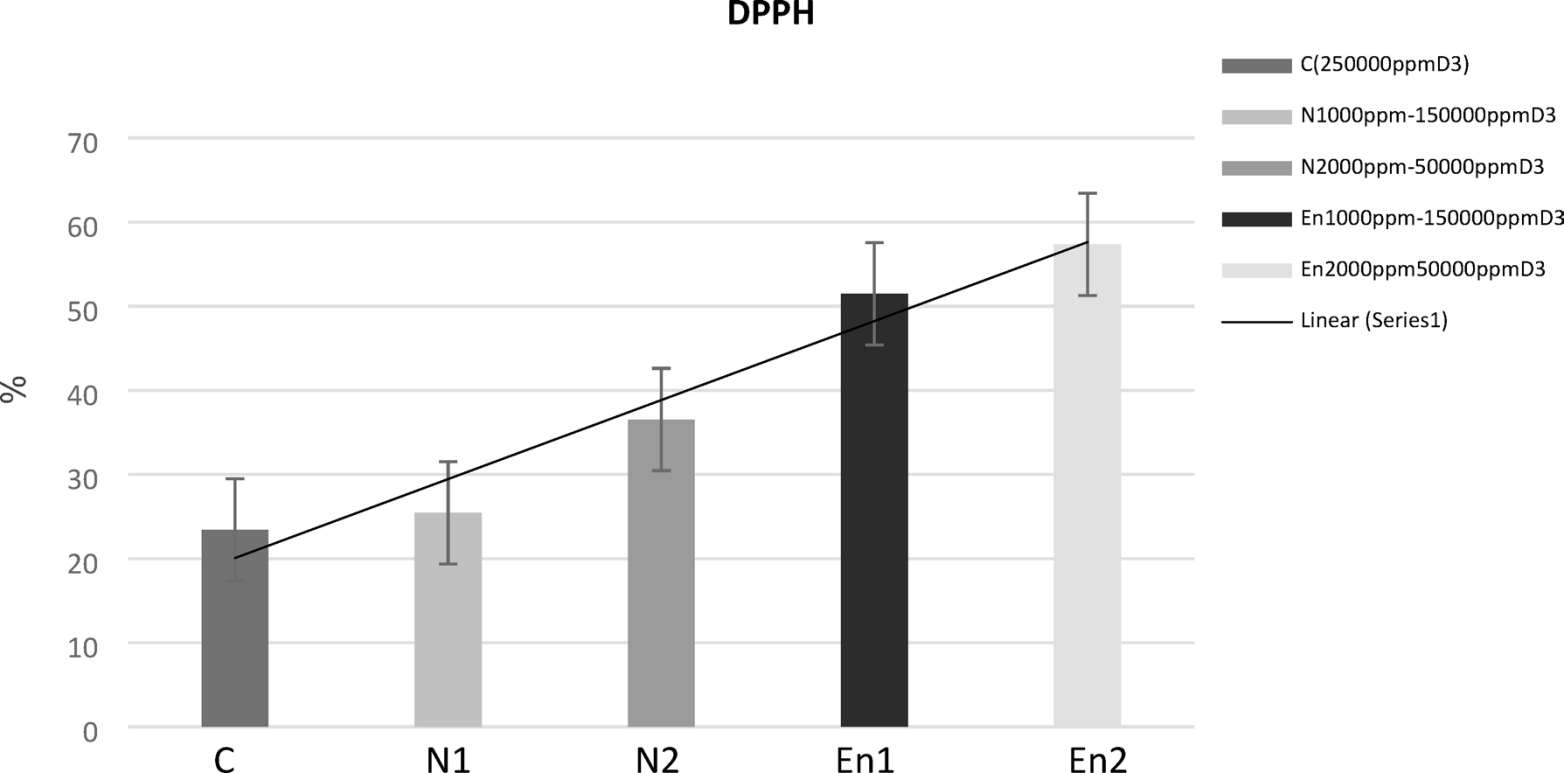
Comparison of DPPH inhibition activity in free and encapsulated film.

The antioxidant activity of the edible films was assessed using the DPPH radical scavenging assay, and the results are illustrated in Fig. 3. Incorporation of CMF peel mucilage significantly enhanced the antioxidant properties of the films. Notably, encapsulated films exhibited stronger antioxidant activity compared to their free counterparts, highlighting the protective effect of encapsulation on bioactive compounds. CMF peel mucilage is rich in phenolic compounds and flavonoids, with a total phenolic content of 218.16 µg/g, which contributes to its free radical scavenging capacity. These compounds neutralize DPPH radicals by donating hydrogen atoms or electrons^65^. This mechanism is essential for inhibiting oxidative degradation in packaged foods, thereby enhancing shelf life and nutritional quality^66^. The incorporation of natural antioxidants such as CMF peel mucilage into edible films aligns with current trends in active food packaging, which aim to slowly release bioactive agents onto the food surface, maintaining efficacy over extended periods^67^. The co-formulation with vitamin D3 in the oil phase further stabilizes antioxidant compounds, protecting them from oxidation and improving the film’s functional properties^68^. Moreover, phenolic acids present in the mucilage exhibit dual functionality, as they not only scavenge free radicals but also exhibit antimicrobial action. They disrupt microbial membranes, increase cytoplasmic permeability to ATP, and ultimately lead to cell death^44^. However, their interaction with food matrix components such as proteins and lipids may reduce their antimicrobial efficacy^45^. Overall, the DPPH assay results confirm the antioxidant potential of CMF peel mucilage-based films and support their application in active packaging systems aimed at extending food shelf life and improving safety.

#### Water vapor permeability

Water vapor permeability is a key parameter in the performance of edible films for food packaging, as it directly affects moisture transfer and shelf-life stability. As shown in Table 8, WVP values differed significantly among treatments (*p* < 0.05). The highest WVP was recorded for the control sample (203.55 ± 0.6 g/h·mm·kPa×10⁶), while the lowest belonged to the EN2 sample (161.41 ± 0.1). This reduction in WVP is attributed to the presence of vitamin D3 in the oil phase, which increases the hydrophobicity of the film. Hydrophobic compounds decrease the number of open pores and reduce the mobility of water molecules through the film matrix. These findings are in line with previous studies highlighting the effectiveness of emulsion-based films in enhancing water barrier properties by adding lipidic components^69^. Moreover, CMF peel mucilage, rich in polar groups, could increase WVP if used alone. However, its combination with hydrophobic agents like vitamin D3 in an encapsulated system results in a more compact polymer network, thus decreasing permeability.

#### Water solubility

Water solubility is an important characteristic in designing biodegradable and water-resistant films. The data presented in Table 8 demonstrate significant differences among treatments (*p* < 0.05). The highest solubility was observed in N2 (46.89 ± 0.01%), indicating that higher concentrations of free mucilage increased the film’s hydrophilicity. In contrast, the lowest solubility values were recorded in EN1 (23.45 ± 0.01%) and EN2 (24.39 ± 0.01%), reflecting the effect of encapsulation on reducing water affinity. The observed decrease in solubility for encapsulated treatments may result from strong intermolecular interactions between carboxymethyl cellulose and the encapsulating agents, such as calcium alginate polymers^70^. These interactions create a tighter polymer matrix, decreasing water penetration and the release of soluble compounds. Studies by others also confirm that the inclusion of hydrophobic substances, such as lipid-based nanoemulsions or fat-soluble vitamins, can reduce the solubility of methylcellulose-based films^71,72^. This phenomenon is further supported by the presence of hydroxyl and carboxyl groups in carboxymethyl cellulose, which promote hydrogen bonding and enhance the structural integrity of the film^73^. These results suggest that the incorporation of CMF peel mucilage in combination with encapsulated vitamin D3 can successfully improve the water resistance and barrier properties of edible films, making them suitable for moisture-sensitive food applications.

**Table 8:** Water vapor permeability and water solubility of Films.

Sample	Name	WVP g/h mm K.pa×10^6^	Water solubility %
(250ppmD_3_) C	C	0. 6^a^ ± 203.55	0.1^c^± 28.93
N100ppm-150ppmD_3_	N1	0.10^c^ ± 178.33	0.1^b^ ± 45.84
N200ppm-50ppmD_3_	N2	0.1^b^ ± 199.65	0.1^a^ ± 46.89
En100ppm-150ppmD_3_	En1	0.1^d^ ± 161.41	0.1^e^ ± 23.45
En200ppm50ppmD_3_	En2	0.16^d^ ± 162.40	0.1^d^ ± 24.39

Different letters in the same column indicate a significant difference among the samples, as revealed by Duncan’s test, *p* < 0.05.

#### Thickness and density

The thickness results presented in Table 9 indicated that N1, N2, EN1, and EN2 did not show significant differences, but all of these treatments exhibited significant differences when compared to the control (C) (*p* < 0.05). The highest thickness was observed in the N1 treatment (0.138 mm), while the lowest thickness was recorded for the control (0.025 mm). This suggests that the addition of mucilage contributed to a significant increase in film thickness.

As for density, there were no significant differences between the treatments, though the highest density was observed in N2 (1.153 g/mm³), and the lowest density was recorded in the control (1.114 g/mm³). This finding indicates that the increase in mucilage concentration did not significantly affect the density of the films, but still resulted in subtle changes in the physical properties. The increase in thickness is attributed to the higher concentration of solid materials (mucilage) in the films. The arrangement of mucilage molecules in the matrix also played a role in increasing the thickness of the films. Controlling film thickness is essential to ensure the physical integrity and barrier properties of the films, as thicker films generally provide better resistance to moisture and mechanical stress^69^. In addition, previous studies have reported that the inclusion of mucilage and nanoemulsions, particularly those enriched with vitamin D3, can enhance the hydrophobicity and structural properties of the films. The presence of mucilage in combination with a nanoemulsion (such as that derived from quince seed) has been shown to increase thickness and hydrophobicity, contributing to better physical performance in food packaging applications^68^.

**Table 9:** Determination of thickness and density in free and encapsulated nanoemulsion films.

Name	Sample	density(g/mm^3^)	thickness(mm)
C	C(250ppmD_3_)	0.11^a^ ± 1.114	0.001^b^ ± 0.025
N1	N100ppm-150ppmD_3_	0.60^a^ ± 1.123	0.015^a^ ± 0.128
N2	N200ppm-50ppmD_3_	0.185^a^ ± 1.153	0.012^a^ ± 0.138
En1	En100ppm-150ppmD_3_	0.2^a^ ± 1.134	0.02^a^ ± 0.124
En2	En200ppm50ppmD_3_	0.2^a^ ± 1.136	0.010^a^ ± 0.131

Different letters in the same column indicate a significant difference among the samples, as revealed by Duncan’s test, *p* < 0.05.

Density and thickness increased significantly in proportion to its concentration by adding mucilage.

#### Stability of vitamin D3

**Fig. 4:**
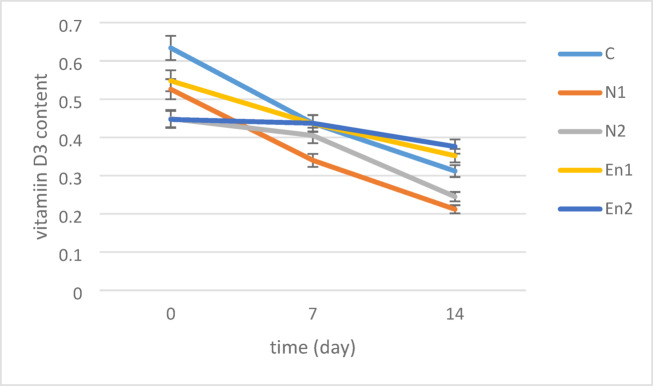
Stability of vitamin D3 during 14-day storage in free and encapsulated nanoemulsion film.

The stability of vitamin D3 in both free and encapsulated nanoemulsion films was evaluated over a period of 0, 7, and 14 days, and the results are shown in Fig. 4. The stability of vitamin D3 significantly decreased with time (*p* < 0.05), showing a steady decline over the 14-day storage period at 25 °C. This finding highlights the sensitivity of vitamin D3 to environmental conditions and emphasizes the need for protective encapsulation in food packaging. On day 0, no significant difference was observed in the stability of vitamin D3 between EN2 and N2 treatments, but a significant difference was found when comparing C and N1 treatments (*p* < 0.05). The highest stability was observed in the control treatment (C) (0.634), while the lowest was recorded for EN2 (0.447). On day 14, the stability of vitamin D3 further decreased, with EN2 maintaining the highest stability (0.376) and N1 showing the lowest stability (0.212). These results confirm that encapsulation significantly improved the stability of vitamin D3 over time, with encapsulated nanoemulsions showing better stability than free nanoemulsions. This improved stability can be attributed to the lipophilic nature of vitamin D3, which allows it to be incorporated into the calcium alginate layer of the encapsulated films. Additionally, the alginate matrix provides a protective barrier that shields the vitamin from degradation caused by exposure to water and other environmental factors^74^. Factors such as heat, moisture, and oxygen can affect the stability of vitamins. As reported by another author, the stability of vitamins is influenced by these environmental factors, and their effectiveness can be altered based on exposure to these elements^75^. In general, the stability of vitamins decreases with higher temperatures, increased rotation speed, and lower moisture content^32^. During storage, vitamin D3 is susceptible to isomerization and decomposition, which can lead to a reduction in its bioactivity^76^. Supporting these findings, the storage stability of vitamin D3 encapsulated in nanoliposomes has been studied, showing that extended storage duration led to reduced degradation and improved stability of vitamin D3 in the nanostructures^77^.

#### FTIR measurement of film

**Fig. 5:**
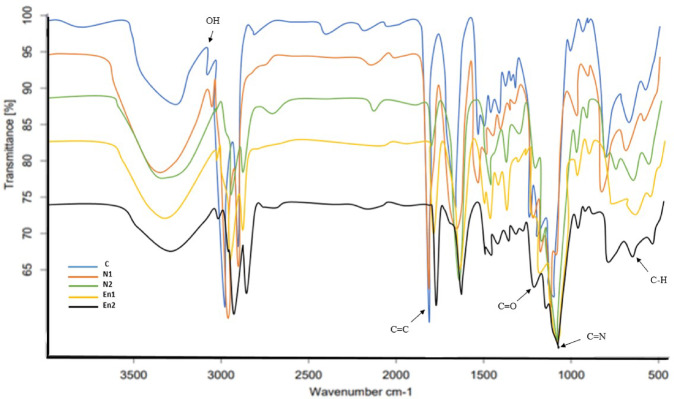
FTIR spectra of film samples containing free and encapsulated nanoemulsions.

As seen in Fig. 5, the peaks in the range of 3000–3600 cm^−^ indicate OH bonds in carboxylic acid and free OH stretching bonds. OH stretching vibrations show intermolecular or intramolecular hydrogen bonds. The bandwidth at 3000–3500 cm^−^ is related to the vibrational stretching of hydroxyl groups, indicating water retention and hydrogen bonding ability in polysaccharides^46^. Peaks in the range of 2800–3000 cm^-1 correspond to NCH and CH2 bending and stretching vibrations of alkanes and alkenes. Typical bands assigned to cellulose in the region of 1630 –900 cm^-1 are visible. The absorption band at 897 cm^-1 is related to ether bonds of glucose units in cellulose. The peak at 1655 cm^-1 is related to the stretching vibrations of the double bond between carbons (C = C). The peak around 1331 cm^-1 may be due to the interaction between the hydroxyl groups of the plasticizer and the film polymers^78^. Samples containing free nanoemulsion showed looser bands in this region, indicating the incorporation of vitamin D3 with the mucilage in the film matrix. These observations showed the greater stability of the added component in the edible film matrix. The characteristic bands at 1597–1600 and 1412–1416 cm^-1 are respectively assigned to COOH carboxyl groups and C-H bending of CH3 stretching vibrations, indicating the presence of vitamin D3 encapsulated with a calcium alginate wall. Peaks at 1036–1044 cm^-1 are attributed to the stretching vibrations of the glycoside bond^79^. Additionally, it has been reported that the 1700 –1600 cm^-1 range is assigned to the amide spectral region and the secondary structure of proteins, which is mainly the result of the stretching of bonds at C = O of the peptide structures. Other peaks at 1020, 2932, and 1238 cm^-1 may be attributed to single carbon-hydrogen and carbon-oxygen bonds. The peak at 1301 cm^-1 is also attributed to the C-O-C stretching vibration bond^80^. The findings of this investigation align with the outcomes of other researchers in their analysis of the FTIR results obtained from CM mucilage. The results of the current study showed that the main component of the prepared film is polysaccharide^10,68^.

#### Thermal *gravimetric analysis (*TGA*)*

**Fig. 6:**
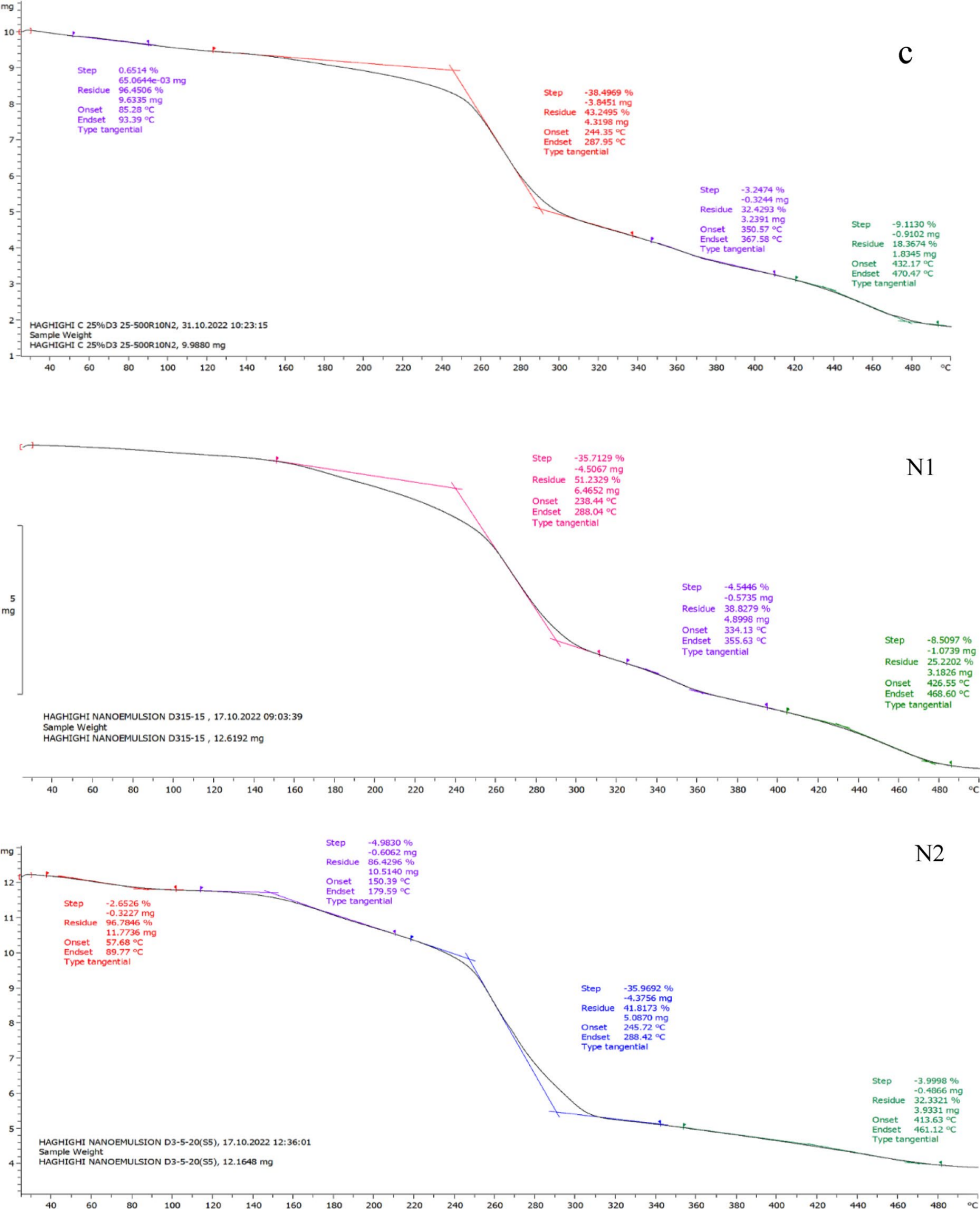

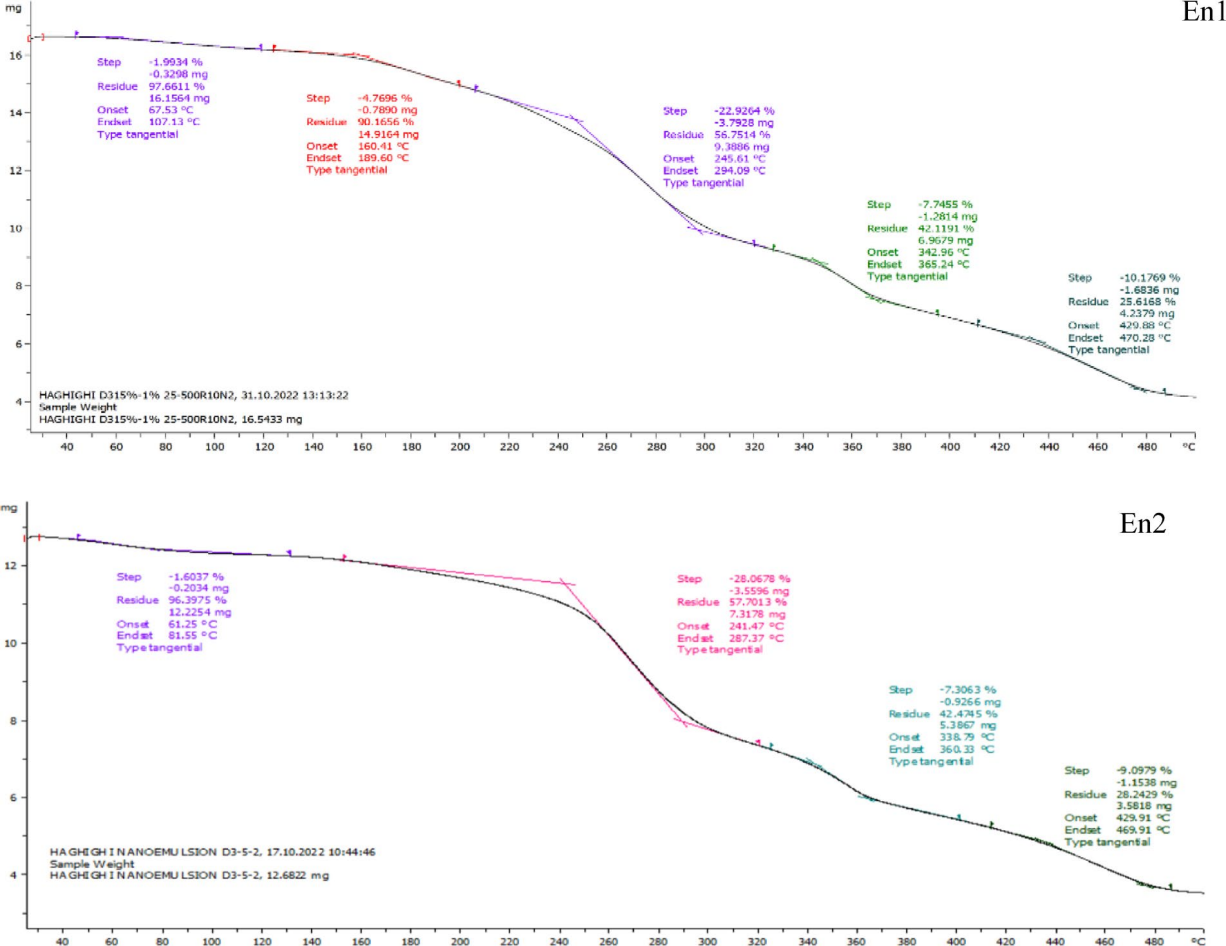
TGA thermogram of free and encapsulated nanoemulsions films.

Thermal gravimetric analysis (TGA) was conducted to evaluate the thermal stability of the films containing free and encapsulated nanoemulsions, and the results are shown in Fig. 6. The TGA curves revealed a multi-stage decomposition process, characteristic of the different phases of thermal degradation, including dehydration, drying, desorption, dissolution, and the destruction of the polymeric structure. At lower temperatures, water-bio-polymer interactions and the water retention capacity of the films are crucial factors influencing the weight loss behavior. The presence of hydrophilic groups in biopolymers enhances the formation of hydrogen bonds with water molecules, increasing the amount of bound water in the film matrix. On the other hand, hydrophobic groups reduce this interaction, leading to lower water content in the films^36^. In the initial phase of the thermal analysis, the TGA curve showed a horizontal plateau, indicating that the sample was pure and free of moisture or impurities. A downward slope at this stage would suggest the presence of impurities, as their mass would decrease with increasing temperature. As the temperature increased, the slope became negative, indicating thermal degradation of the polymer matrix. The first significant weight loss was observed around 250 °C, which can be attributed to the destruction of the polymer structure. In the temperature range of 160–250 °C, samples C, N1, and N2 showed weight loss due to the removal of water or solvents, with approximately 5% weight loss. At 300 °C, the samples exhibited a substantial weight loss (about 40%), attributed to the destruction of the polymeric structure. Between 200 and 300 °C, the carboxymethyl cellulose and calcium alginate matrices began to decompose, leading to a severe weight loss (~ 50%). From 300 °C onwards, further degradation was influenced by the vitamin D3 content in the films. Encapsulation with calcium alginate improved the thermal stability of the films compared to free nanoemulsions. The first weight loss in the encapsulated films occurred around 130 °C, which corresponds to the loss of water from the calcium alginate encapsulation. A second weight loss was observed between 200 and 330 °C, which can be attributed to the pyrolysis of calcium alginate. Finally, by 490 °C, approximately 28.88% of the initial mass remained, suggesting that the encapsulation process significantly enhanced the thermal stability of the nanoemulsion-loaded films. The TGA thermal curve is also useful for studying the stoichiometry or kinetics of a reaction, and the results showed that the degradation of the sample occurred in a single step, with relatively stable intermediates^81^. In comparison, some studies showed no significant effect of henna extract on the thermal properties of films, indicating that certain natural extracts might not influence the thermal behavior of the films significantly^82^. On the other hand, vitamin D3 has been reported to be highly sensitive to thermal degradation at 300 °C, with a loss of approximately 50% of its content, suggesting its instability under such conditions^83^. Similarly, CM nanocapsules have been found to exhibit a lower melting point, with the encapsulated CMF mucilage melting at 383.87 °C, indicating the protective role of the alginate wall in safeguarding the bioactive compounds^84^. Overall, the incorporation of nanoemulsions into the calcium alginate matrix has proven to be an effective strategy in enhancing the thermal stability of the edible films, demonstrating the critical role of formulation strategies in optimizing the performance of these films for food packaging applications.

## Conclusion

This study successfully developed antimicrobial active packaging films using CMF peel mucilage and vitamin D3 nanoemulsions in both free and encapsulated forms. Encapsulation significantly improved the films’ resistance to moisture and heat, enhanced their antioxidant activity, and ensured better stability of the active compounds. The incorporation of natural emulsions into the film matrix was confirmed through FTIR, while TGA results highlighted the superior thermal resistance of the encapsulated systems. The films also demonstrated effective antimicrobial properties, making them promising candidates for active food packaging. Although bioactive packaging is currently more costly, it reflects a step toward a future where food protection is not only efficient but also environmentally responsible. By embracing nature-based materials and innovative delivery systems, this work opens a new window for designing multifunctional, sustainable packaging solutions. Future studies may pave the way for commercial scalability and broader application across food industries.

## Data Availability

The data used to support the findings of this study are available from the corresponding author upon request.
